# Characterization of Chromosomal Instability in Glioblastoma

**DOI:** 10.3389/fgene.2021.810793

**Published:** 2022-01-28

**Authors:** Elisa Balzano, Elena Di Tommaso, Antonio Antoccia, Franca Pelliccia, Simona Giunta

**Affiliations:** ^1^ Laboratory of Molecular Cytogenetics, Dipartimento di Biologia e Biotecnologie “Charles Darwin”, Sapienza Università di Roma, Roma, Italy; ^2^ Laboratory of Genome Evolution, Dipartimento di Biologia e Biotecnologie “Charles Darwin”, Sapienza Università di Roma, Roma, Italy; ^3^ Laboratory of Genetics and Cytogenetics, Dipartimento di Scienze, Università Degli Studi Roma Tre, Roma, Italy

**Keywords:** glioblastoma, cancer, chromosome instability, replicative stress, fragile sites

## Abstract

Glioblastoma multiforme (GBM) is a malignant tumor of the central nervous system (CNS). The poor prognosis of GBM due to resistance to therapy has been associated with high chromosomal instability (CIN). Replication stress is a major cause of CIN that manifests as chromosome rearrangements, fragility, and breaks, including those cytologically expressed within specific chromosome regions named common fragile sites (CFSs). In this work, we characterized the expression of human CFSs in the glioblastoma U-251 MG cell line upon treatment with the inhibitor of DNA polymerase alpha aphidicolin (APH). We observed 52 gaps/breaks located within previously characterized CFSs. We found 17 to be CFSs in GBM cells upon treatment with APH, showing a frequency equal to at least 1% of the total gaps/breaks. We report that two CFSs localized to regions FRA2E (2p13/p12) and FRA2F (2q22) were only found in U-251 MG cells, but not lymphocytes or fibroblasts, after APH treatment. Notably, these glioblastoma-specific CFSs had a relatively high expression compared to the other CFSs with breakage frequency between ∼7 and 9%. Presence of long genes, incomplete replication, and delayed DNA synthesis during mitosis (MiDAS) after APH treatment suggest that an impaired replication process may contribute to this loci-specific fragility in U-251 MG cells. Altogether, our work offers a characterization of common fragile site expression in glioblastoma U-251 MG cells that may be further exploited for cytogenetic and clinical studies to advance our understanding of this incurable cancer.

## Introduction

Fragile sites are defined as gaps/breaks induced by replication stress that are visible on metaphase chromosomes. The Human Genome database currently reports 120 chromosomal regions to be fragile sites of which 30 are classified as rare fragile sites (RFSs) and 90 as common fragile sites (CFSs) (Feng and Chakraborty, 2018). To be considered fragile sites, these loci must exhibit as a chromosomal gap/break in at least 1% out of all gaps/breaks upon replication stress induced by treatments such as the DNA polymerase alpha inhibitor, aphidicolin (APH) (Le Tallec et al., 2011; Le Tallec et al., 2013; Arlt et al., 2003). The expression of common fragile sites as chromosome gaps/breaks may not be triggered by a single factor but rather by a combination of different mechanisms. Several studies in the last decade have added to our understanding of which CFS molecular features contribute to their fragility, especially regarding the convergence of replication and transcription machineries within these loci (Helmrich et al., 2011). Growing evidence shows that CFS instability can vary between different cell types in response to replicative stress conditions, partly due to tissue-specific expression of genes located within each CFS (Debatisse et al., 2012). Similarly, transcription of non-coding RNAs can also trigger chromosomal fragility within CFSs as has been shown for other fragile regions of the genome such as centromeres (Balzano et al., 2021; Balzano and Giunta, 2020; Giunta, 2018), leading to rearrangements and aneuploidy (Giunta et al., 2021; Giunta and Funabiki, 2017). Indeed, many of the common fragile sites recorded in lymphocytes harbor genes longer than 650 kb, called very long genes (VLGs) (Smith et al., 2006; Bosco et al., 2010) and long–non-coding RNAs (lncRNAs) as we recently characterized in fibroblasts (Maccaroni et al., 2020). A connection between the level of transcription and the frequency of instability of the corresponding region has been reported (Helmrich et al., 2011; Brison et al., 2019). However, APH delays replication timing (RT) of large genes in either fragile or non-fragile loci, highlighting that delayed replication timing and transcription processes alone are insufficient to drive CFS fragility (Sarni et al., 2020). Recent evidence shows that CFSs are chromatin regions with a defective condensin loading due to an under-replicated state which persists until mitosis (Boteva et al., 2020). These faulty chromatin-folding regions have been detected as sites of mitotic DNA synthesis (MiDAS) (Ji et al., 2020; Macheret et al., 2020), implying that chromatin conformation and delayed replication further underline their fragility (Miotto et al., 2016).

Fragile sites represent threats to genome stability as breaks on metaphase chromosomes but also as regions that are hot spots for deletions of tumor suppressor genes (TSGs) in cancer cells (Casper et al., 2002; Smith et al., 2007; Sarni and Kerem, 2016). The degree of a cause–effect relationship between the expression of fragile sites and TSG-driven transformation is unclear. However, mapping these fragile regions throughout the human genome in several tissues might identify specific tumor-associated stress sites that can contribute to malignancy.

To this end, we used the glioblastoma multiforme U-251 MG cell line as a genetic model in which we characterized common fragile site expression under mild replication stress using the DNA polymerase alpha inhibitor, aphidicolin (APH). We utilized low doses of APH to analyze its effects on metaphases and interphase nuclei and obtained a detailed quantification of CFS expression by scoring for breakage frequency across all known human CFS chromosomal regions. As a control, we compared GBM cells against lymphocytes taken from peripheral blood of healthy individuals and fibroblasts previously used in the study by Maccaroni et al., 2020 to analyze the tissue-specific responses to APH treatment. We found two glioblastoma-specific CFSs along with several CFSs that are expressed in GBM cells at a higher frequency than in primary tissues. We observed presence of long genes and delayed replication within some of these fragile regions, likely contributing to their breakage and expression as CFSs. Future studies of these CFSs can offer a window of opportunity to better understand CIN in GBM cells and may inform novel cancer treatments, such as use of transcription or DNA damage response (DDR) inhibitors to prevent clone selection, evolution, progression, and resistance of this deadly tumor.

## Materials and Methods

### Human Cell Cultures

The human glioblastoma U-251 MG cell line (Astrocytoma IV WHO grade) was purchased from Banca Biologica and Cell Factory (Banca Biologica and Cell Factory, Genoa, Italy) and was provided by A.A.; U-251 MG cells were grown in Eagle’s Minimum Essential Medium (EMEM; Euroclone) supplemented with 10% fetal bovine serum (FBS) (Corning), 1% penicillin (Thermo Fisher Scientific), and 1% L-glutamine (Thermo Fisher Scientific) at 37°C with 5% CO_2_. Lymphocyte cultures were prepared from human peripheral whole blood of healthy individuals, collected with heparin; cells were grown in RPMI medium (Corning) supplemented with 10% FBS, 1% penicillin, and 1% L-glutamine at 37°C with 5% CO_2_. For stimulation of T lymphocyte proliferation, phytohemagglutinin (PHA, GIBCO, 3%) was added to the culture medium for 72 h. To induce common fragile sites, aphidicolin (0.4 μM) was added to the medium of both lymphocytes and U-251 MG cells for 22–24 h. To collect the mitotic cells, colchicine (Sigma-Aldrich) was supplemented to the medium of lymphocytes (1 mM) and U-251 MG cells (5 µM) for 2 and 4 h, respectively. For replication timing analysis, 5-Bromo-2′-deoxyuridine (BrdU; 10 μM) was added 20 min prior to harvesting the cells.

### Metaphase Spreads Preparation

Upon colchicine treatment, lymphocytes and U-251 MG cells were harvested for metaphase spreads preparation. U-251 MG cells were trypsinized (trypsin 0.1% EDTA, Corning) and centrifuged prior to the addition of hypotonic solution (KCl 0.075 M) for 20 min at 37°C. The hypotonic treatment was performed for 8 min in lymphocytes; after centrifugation, Ibraimov’s solution (3% methanol and 5% acetic acid in dH_2_O) was used to remove the erythrocytes. Swollen cells were centrifuged twice and resuspended in cold fixative solution (methanol:acetic acid at a ratio of 3:1) and then stored overnight at −20°C. The metaphase spreads and interphasic nuclei were dropped onto clean glass slides and air-dried. The slides were stored at 4°C until subsequent analysis.

### Cytogenetic Observation and Analysis

The slides were stained with 4% Giemsa (Carlo Erba) to detect chromosome aberrations and then with Chromomycin A3 (R-banding) to localize gaps/breaks, according to ISNC recommendation (Maccaroni et al., 2020). The karyotype of this glioblastoma multiforme U-251 MG clone using mFISH (multicolor-FISH) was performed by Antoccia Laboratory as can be seen in the study by Berardinelli et al., 2018. The quantification of chromosome aberrations was done according to the OECD guideline (OECD Test No, 2016).

### BAC Extraction and Labeling by Nick Translation

The bacterial artificial chromosomes (BACs) were chosen from NCBI GenBank, 2121 (https://www.ncbi.nlm.nih.gov/genbank/) for chromosome 1 (RP11-316C12, chr1: 71,385,313-71,476,945), chromosome 3 (RP11-324H4 chr3: 116,954,325-117,125,019), and chromosome 7 (RP11 321C7 chr7: 67,705,408 - 67,771,498). Bacteria were grown in 10 ml of Luria-Bertani (LB) medium and selected with chloramphenicol (20 μg/ml). BACs were extracted by alkaline lysis and subsequently labeled by Nick Translation with bio-16-dUTP (biotin-16-deoxy-Uridine Triphosphate) and/or dig-16-dUTP (digoxigenin-16-deoxy-Uridine Triphosphate). The labeled probes were used for fluorescent *in situ* hybridization (FISH) experiments on interphase nuclei.

### DNA Fluorescence *in situ* Hybridization

The slides were treated with RNase (100 µg/ml in 2× saline sodium citrate SSC solution) for 1 h at 37°C and dehydrated by washing for 5 min in 70, 90, and 100% ethanol. After air-drying, the slides were aged at 65°C for 60 min and denatured at 80°C for 2 min with 70% formamide (Sigma) in 2× SSC. The denaturation was stopped with cold 70% ethanol for 5 min, and the slides were dehydrated again with 90 and 100% ethanol and air-dried prior to hybridization using the denatured probes (200 ng). The used BACs were RP11-316C12 (1p31.1), RP11-324H4 (3q13.3), and RP11 321C7 (7q11.2). Sequentially, the overnight BAC probe incubation at 37°C and 3 × 5-min post-hybridization washes with 1× SSC were performed at 60°C. The slides were then incubated for 30 min with anti-digoxigenin-rhodamine antibody (1:20, Roche). Three washes with 0.1% Tween20 in 2× SSC were performed. The slides were counterstained with DAPI (4′,6′-diamidino-2-phenylindole hydrochloride, Sigma; 1 μg/ml), diluted 1:300 in VECTASHIELD Antifade Mounting Medium (Vector Laboratories).

### Immunofluorescence on Interphase Nuclei

Immunofluorescence (IF) against BrdU was performed to distinguish the different stages of the S-phase. The slides were incubated for 1 h at room temperature with the anti-mouse BrdU monoclonal antibody (MoBU-1, Thermo Fisher Scientific), diluted 1:300 in 5% FBS in 1X PBS, pH 7.4. After 3 x 5-min washes in 1X PBS, the slides were incubated with FITC-conjugated anti-mouse IgG H&L antibody (Abcam) (1:1000 in 1X PBS, pH 7.4) for 1 h at room temperature. After 4 x 5-min washes in 1X PBS, a DAPI:VECTASHIELD Antifade solution (1: 300) was used to mount the slides.

### Acquisition and Processing of Sample Observations

Metaphase spreads and nuclei were observed at a magnification of 100X using an epifluorescence microscope (Zeiss Axioplan) equipped with a CCD camera (charge-coupled device). The images of metaphase spreads and nuclei were taken using RSImage software and then processed using Photoshop (Adobe) software.

### Fragile Sites Sequence Analysis

In addition to fragile site expression, we analyzed the sequence and gene composition for the region using three Human Genome Resources (NCBI Genome Data Viewerm, 2021: https://www.ncbi.nlm.nih.gov/genome/gdv/ [Release Data May 16, 2021]; Ensembl, 2021: http://www.ensembl.org/Homo_sapiens/Location/Genome [Human GRCh38p13]; GeneCards, 2021: https://www.genecards.org/). The characterization is visible in Supplementary Table S1.

The elements within the fragile sites characterized include location, length, and gene expression. Figure 6 and Supplementary Figure S2 represent ideograms for the chosen fragile sites with representations of the cytogenetic band and the genes expressed in brain tissue.

### Statistical Analysis

Statistical paired *t*-tests were calculated on Prism (TablePad software). Individual *p* values are indicated in the figures. *p* values: ns (not significant) *p* > 0.05, *p ≤ 0.05, **p < 0.01, ***p < 0.001, and ****p < 0.0001.

## Results

### Replication Stress Induces Chromosomal Instability in the U-251 MG Glioblastoma Cell Line

A complete and accurate DNA replication process is essential for chromosome integrity. In cancer, DNA synthesis can be compromised by a lack of checkpoint control leading to mitotic arrest and/or genome instability.

**FIGURE 1:**
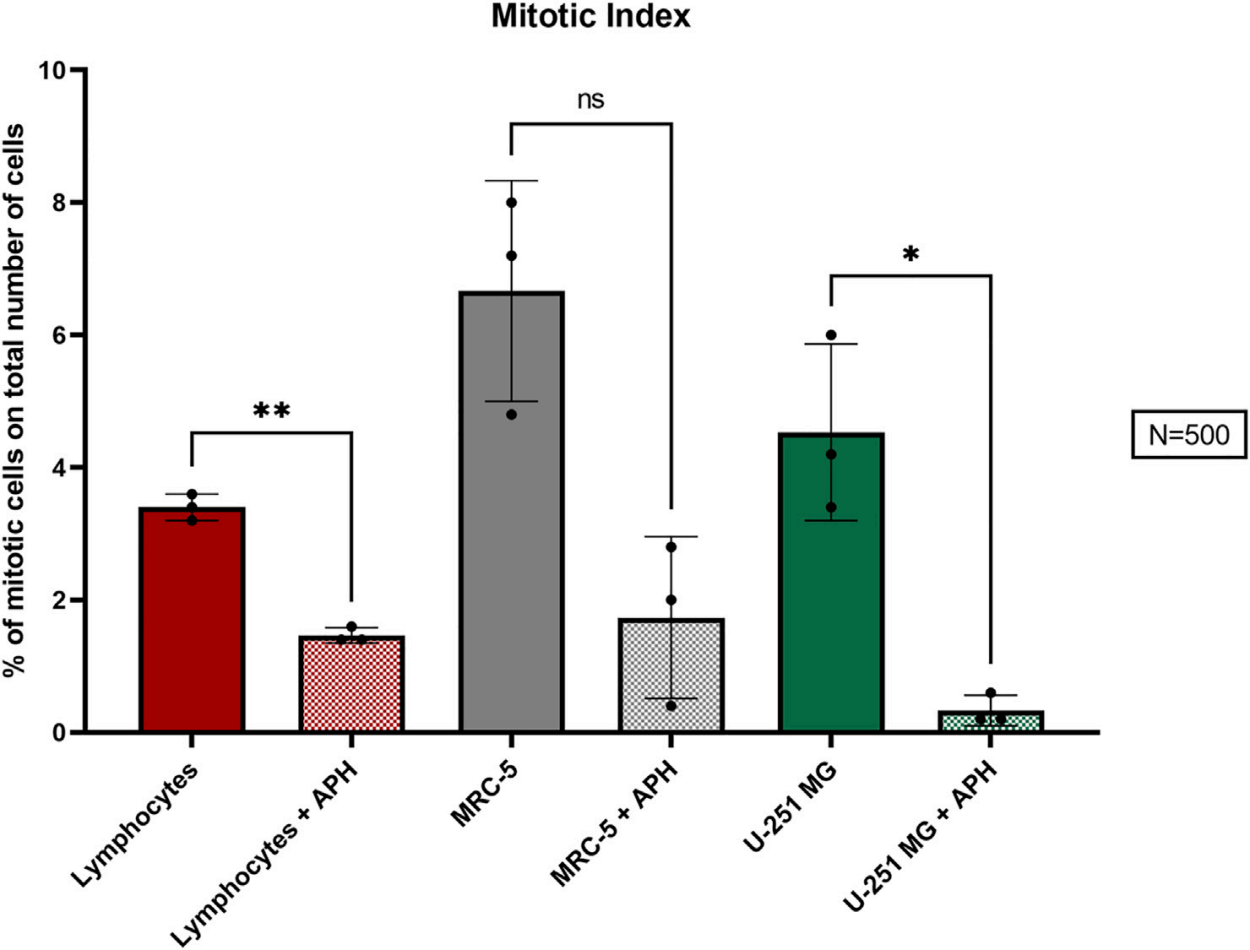
Percentage of the M.I. (mitotic index) in the three cell types, lymphocytes (red), MRC-5 fibroblasts (gray), and U-251 MG GBM cells (green) in control and APH-treated conditions. The color coding of the three cell types is maintained throughout the figures. The error bars represent the standard deviation (SD) determined from 3 independent experiments (*N* = 500 cells for each replicate). Paired *t*-test was used to calculate the *p* values, where *p* > 0.05 ns, *p ≤ 0.05, **p < 0.01, ***p < 0.001, and ****p < 0.0001.

To understand how replication stress affects GBM cells, we evaluated the mitotic index of U-251 MG glioblastoma cells compared with primary lymphocytes and MRC-5 fibroblasts upon treatment with a low dose of APH (0.4 μM) for 24 h. We quantified the ratio of mitotic cells on the total of 500 cells and found that aphidicolin-treated cells show a significant reduction in the mitotic index (M.I.) of U-251 MG glioblastoma cells similarly to lymphocytes (Figure 1).

We further assessed a variety of cellular phenotypes in both metaphase spreads and in interphase nuclei such as blebbing, cytoplasmic bridges, and micronuclei (Supplementary Figures S1A–C). Comparing the response under the APH stress condition in all three cell types, we found a trend of increased cytoplasmic bridges (Supplementary Figure S1B). Instead, the frequency of cellular blebbing and micronuclei remained unaffected between untreated and treated conditions in glioblastoma, lymphocytes, and fibroblasts (Supplementary Figures S1A,C). These results suggest that cells are likely arrested before entry into mitosis or after chromosome segregation in G1 after replication stress or that low-dose APH triggers only very mild phenotypes.

**FIGURE 2:**
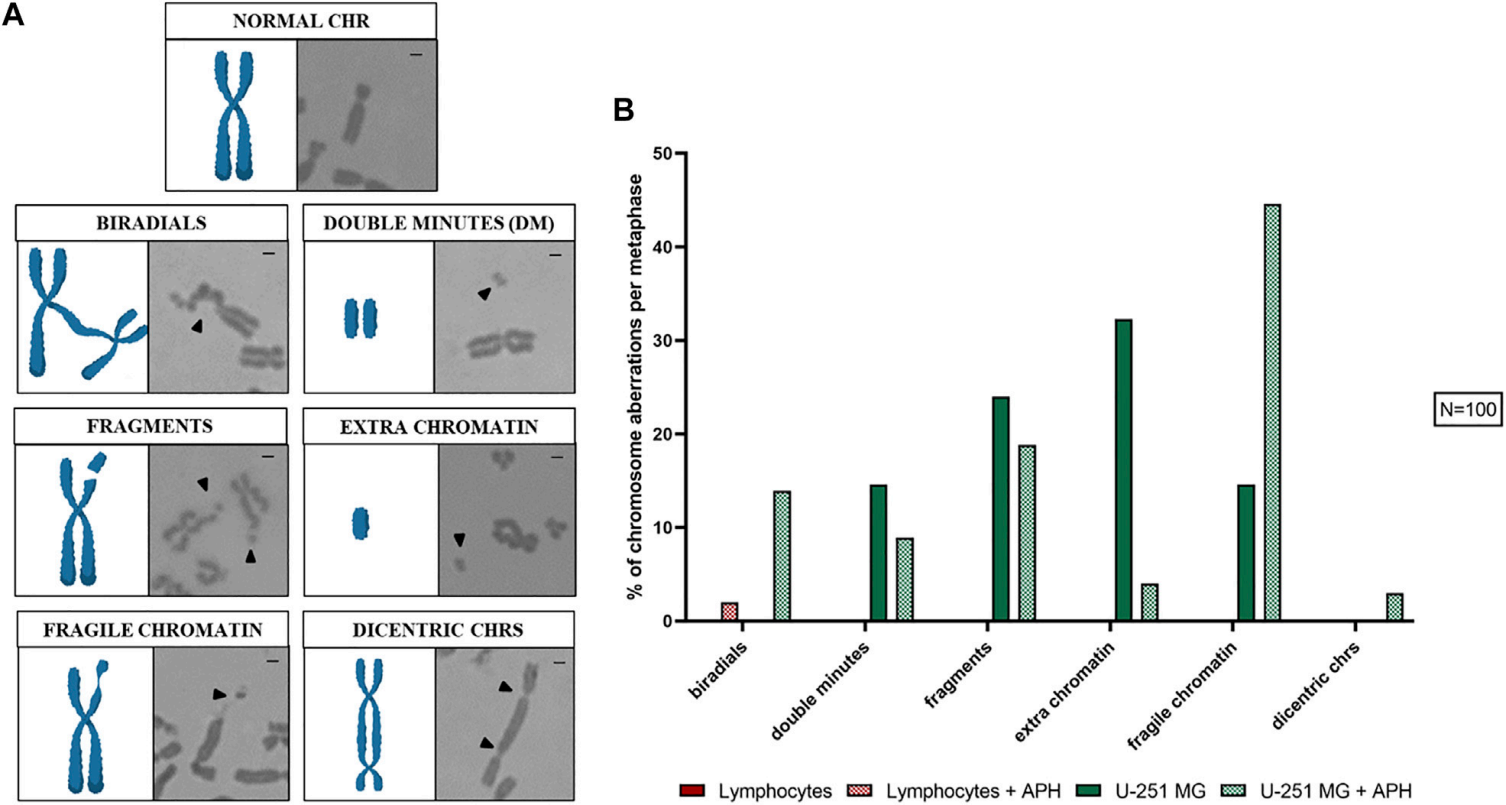
Chromosome aberrations found in U-251 MG metaphases are represented **(A)**, a cartoon model (left panel) and Giemsa staining (right panel) can be seen for each aberration scored: normal chr (chromosome), biradials, double minutes (DM), fragments, extra chromatin, fragile chromatin, and dicentric chrs (chromosomes). In the graph **(B)**, the average number of chromosome aberrations was counted per metaphase. Number of metaphases counted for one replicate was 100 for each control and APH-treated condition (*N* = 100). Scale bar: 1 µm.

The effect of APH in mitotic cells was evaluated on 100 metaphases, where we scored chromosome aberrations (Figure 2A) such as biradials, double minutes (DMs), fragments, extra-chromatin, fragile chromatin, and dicentric chromosomes. As expected, we found several of these phenotypes to be present in untreated glioblastoma cells that were absent in lymphocytes. Interestingly, APH treatment caused an increase in the total amount of chromosome aberrations only in glioblastoma but not in lymphocytes, including biradials, fragile chromatin, and dicentric chromosomes (Figure 2B, graph). Conversely, some aberrations decreased upon APH treatment, such as DMs, DNA fragments, and extra chromatin, suggesting potential activation of checkpoints or repair upon replication stress under these conditions in U-251 MG glioblastoma cells.

**FIGURE 3:**
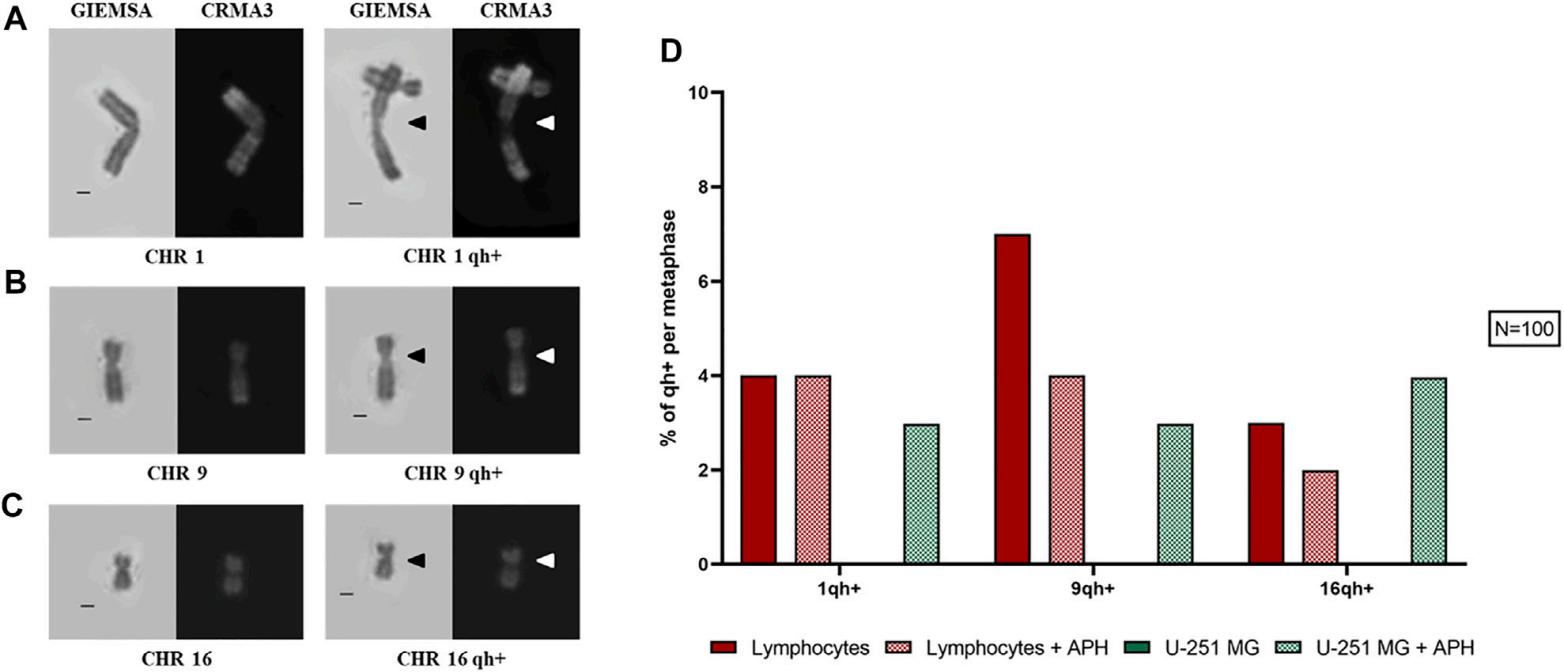
Left: sub-condensed pericentromeric regions (qh+) on chromosome 1 **(A)**, 9 **(B),** and 16 **(C)**. Right: percentage of each qh+ on chromosome 1, 9, and 16 in both lymphocytes and glioblastoma cells under the control and low APH conditions **(D)**. Black arrows indicate qh+ in Giemsa staining and white arrow the qh+ in Chromomycin A3 staining. Number of counted metaphases under control and APH-treated conditions was 100 (*N* = 100) for one replicate. CHR, chromosome. Scale bar: 1 µm.

On 100 metaphase spreads, we also analyzed the pericentromeric heterochromatin for chromosomes 1, 9, and 16 being present as a morphological variant in a sub-condensed state named “qh+,” visible similarly to a secondary constriction (Figures 3A–C). In the human karyotype, these are the only chromosomes, including the Y chromosome, that exhibit this structural peculiarity (Purandare et al., 2011; Sipek Jr et al., 2014). In the case of glioblastoma cells, under-condensed pericentromeric chromatin was highly increased only upon treatment with APH. In lymphocytes, instead, we observed a similar qh+ expression under both untreated and APH conditions, except for chromosome 9 (Figure 3D, graph).

Altogether, our data show that GBM cells are highly affected by replication stressors even low-dose APH, leading to a decrease in overall number of mitoses and specific fragile chromosome phenotypes implying chromatin fragility.

### Glioblastoma-Specific Expression of Common Fragile Sites After Replication Stress

**FIGURE 4:**
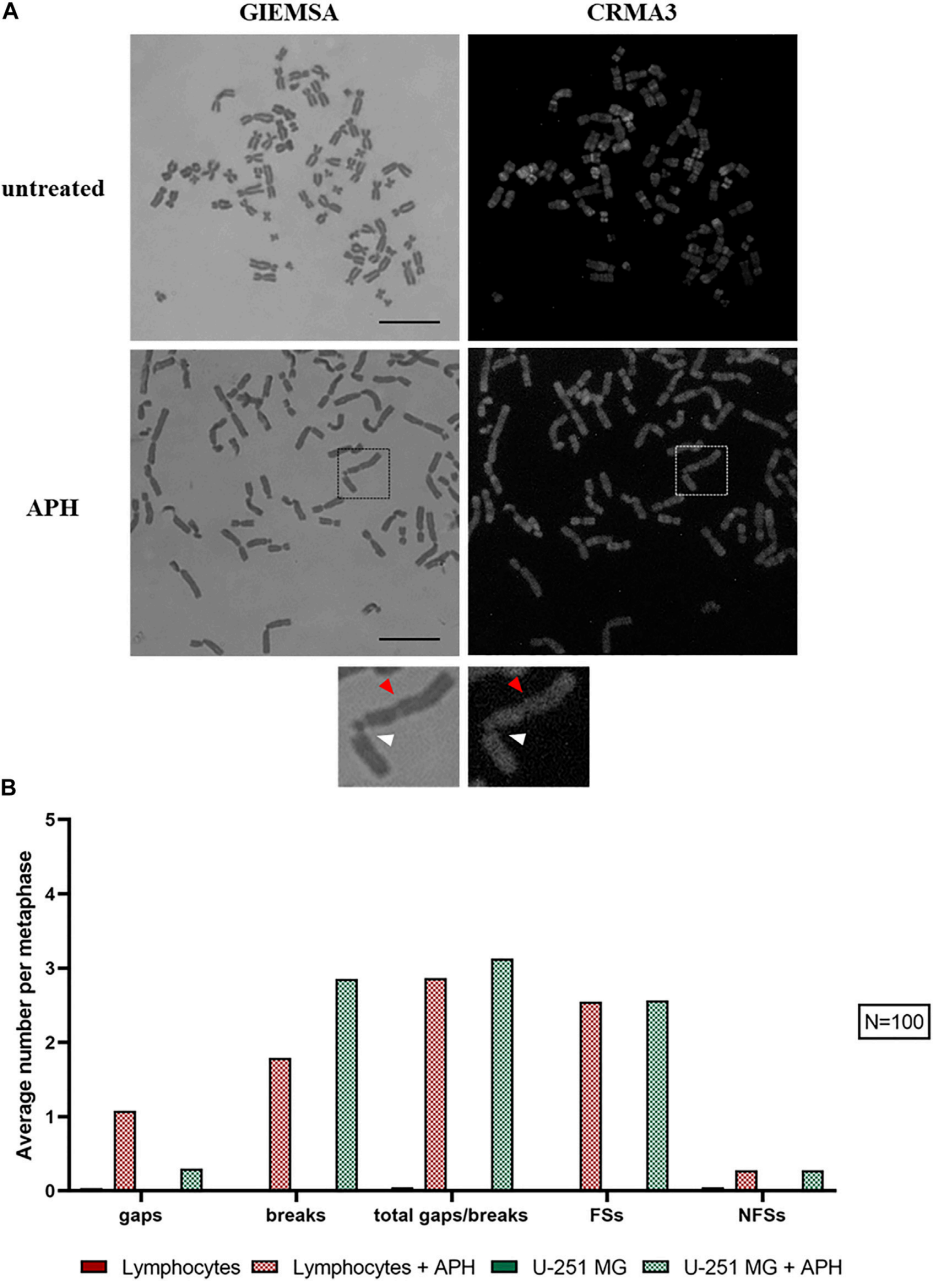
U-251 MG metaphases stained with Giemsa and CRMA3 in control and APH-treated conditions; the brackets highlight chromosome 2, where white arrows indicate the break within FRA2E (2p12/13) and red arrows the gap within FRA2F (2q22) **(A)**. The graph shows the average number of gaps/breaks per metaphase under both conditions and how many of these lesions were fragile sites (FSs) or non-fragile sites (NFSs) **(B)**. Number of counted metaphases under control and APH-treated conditions was 100 (*N* = 100) for one replicate. Scale bar: 10 µm.

**FIGURE 5:**
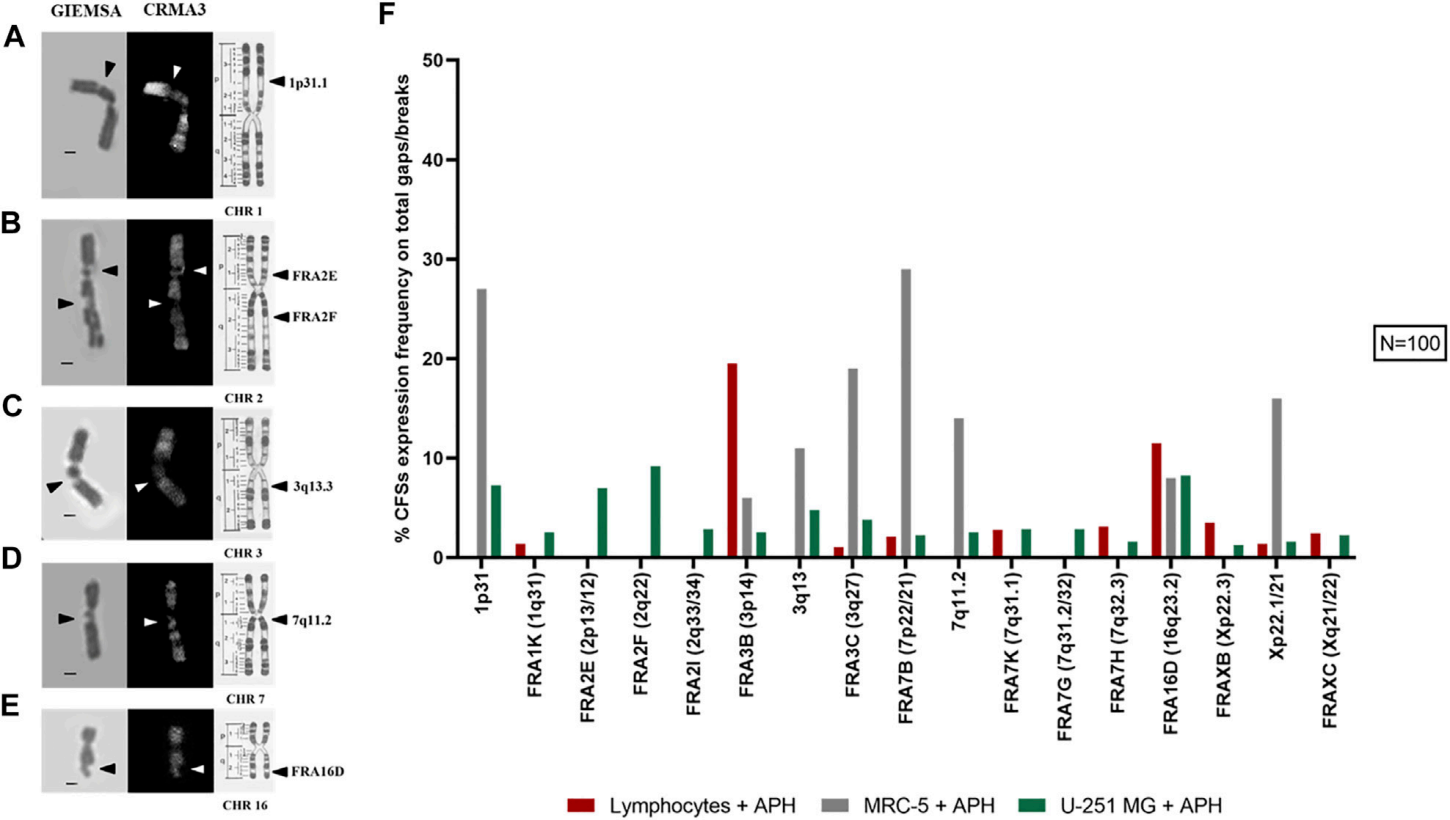
Example images of the most expressed CFSs in U-251 MG cells are shown: 1p31.1 **(A)**, FRA2E (2p13/12) and FRA2F (2q22) **(B)**, 3q13.3 **(C)**, 7q11.2 **(D)**, and FRA16D (16q23.2) **(E)**. R-banding by CRMA3 staining allows the recognition of the cytological band where the lesion is localized (chromosome ideograms are modified from the study by Dutrillaux et al., 1976). In the graph **(F)**, we see CFSs expressed under APH treatment with an expression frequency equal to at least 1% of the total number of gaps/breaks in lymphocytes, fibroblasts, and glioblastoma cells. Number of metaphases counted for control and APH-treated conditions was 100 (*N* = 100) for one replicate. CHR, chromosome. Scale bar: 1 µm.

Next, we investigated the incidence of DNA gaps/breaks under normal and replication stress conditions, and we compared the occurrence of common fragile sites. We used two stains, Giemsa and CRMA3 (Chromomycin A3), on the same metaphase to recognize both expression of the site and the specific cytogenetic band involved in the gap/break (Figure 4A). Notably, gaps and breaks were mainly detected after APH treatment within fragile sites in both U-251 MG cells and in lymphocytes (Figure 4B, graph). Cytogenetic observation of over 100 metaphases enabled us to detect and map gaps/breaks to CFSs on glioblastoma metaphase chromosomes. We scored all known human CFSs across primary cells (lymphocytes and MRC-5 fibroblasts) and GBM cells (Figure 5F, graph). We found 52 CFSs expressed as gaps/breaks in GBM cells; among them, only 17 CFSs showed fragility with a frequency equal to at least 1% on the total of gaps/breaks upon APH treatment. Importantly, two of these CFSs localized to regions FRA2E (2p13/p12) and FRA2F (2q22) appeared to be glioblastoma-specific since the breaks/gaps induced by APH were only seen in U-251 MG cells (Figure 5B) and were not found in lymphocytes or fibroblasts under these experimental conditions.

In line with previous evidence, our data show the striking heterogeneity in expressing specific CFSs at different frequencies across the three cell types. For all cells, the fragile sites did not occur without replicative stress. After APH is supplemented to the cell culture medium, high expression of specific fragile sites such as FRA2E and FRA2F (Figure 5B) were found in the glioblastoma cell line.

The effect of APH in promoting expression varies in frequency between CFSs observed in lymphocytes and in U-251 MG cells. For instance, the FRA3B site in lymphocytes has an expression greater than 19%, while in GBM cells it is less than 3% (Figure 5F, graph). Conversely, glioblastoma-specific breaks localized to regions FRA2E (2p13/p12) and FRA2F (2q22) were only seen in U-251 MG cells and were not observed in either lymphocytes or fibroblasts.

FRA16D (Figure 5E) appears as a CFS in all three cell types, potentially underscoring a different mechanism of fragility of this region that is not tissue-specific. We also identified several characteristic fibroblasts sites, such as 1p31.1 (Figure 5A), 3q13.3 (Figure 5C), and 7q11.2 (Figure 5D) as previously shown (Murano et al., 1989; Le Tallec et al., 2011; Maccaroni et al., 2020), which were also expressed as gaps and breaks in GBM (Figure 5F, graph).

Altogether, our data show that CFSs have a very different expression frequency in different cell types underscoring the tissue-specific expression of CFSs (Le Tallec et al., 2011; Maccaroni et al., 2020) and pointing to potential glioblastoma-specific vulnerability upon replication stress within CFSs FRA2E (2p13/p12) and FRA2F (2q22) seen in U-251 MG cells.

**FIGURE 6:**
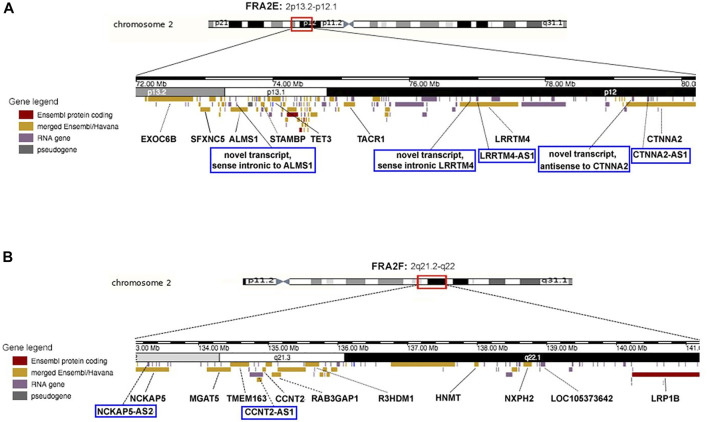
Schematic representation of the most expressed fragile sites in glioblastoma cells: FRA2E (2p13/p12) **(A)** and FRA2F (2q22) **(B)**. In every ideogram, only the genes that are primarily expressed in the brain are indicated. Colors correspond to different genes localized in these specific regions (delimitated by the red brackets). Highlighted genes in blue brackets are the antisense RNAs (asRNAs) and intronic RNAs (itRNAs).

Given the specificity of the CFS FRA2E and FRA2F expression in GBM, we analyzed the sequence composition and the presence of transcripts of these regions. A detailed characterization was obtained by using different databases (NCBI, Ensembl, and GeneCards) (Supplementary Table S1). We found evidence of long genes that are frequently expressed in brain tissue (for FRA2E and FRA2F see Figure 6; for 1p31.1, 3q13.3, and 7q11.2, see Supplementary Figure S2). Interestingly, in addition to several long transcriptionally active genes in the brain, we also observed many antisense and intronic RNA transcripts that we hypothesize may also contribute to promoting glioblastoma-specific chromosomal fragility.

### CFSs Replication Timing Analysis

**FIGURE 7:**
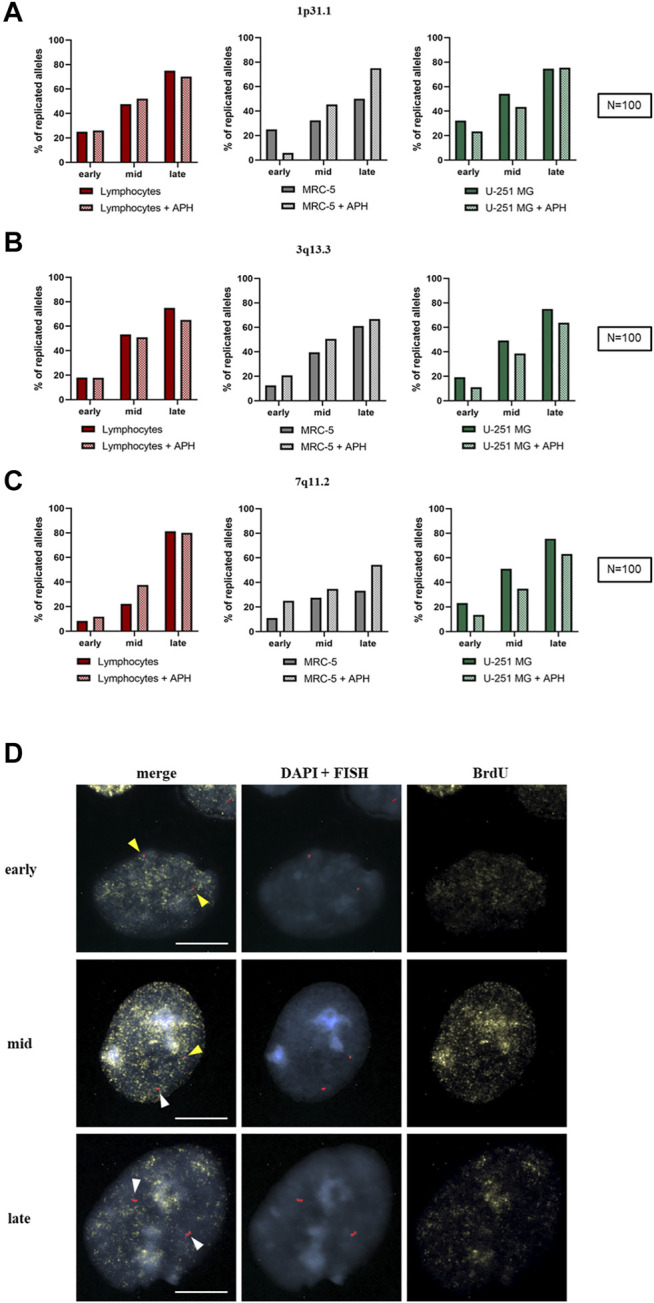
Replication timing of three CFSs in lymphocytes (red), MRC-5 fibroblasts (gray), and U-251 MG GBM cells (green). The graph represents the replicated alleles in each substage of the S-phase for 1p31.1 **(A)**, 3q13.3 **(B)**, and 7q11.2 **(C)**. Example of nuclei stained with DAPI (blue), FISH spots for 3q13.3 with probe RP11-324H4 (red), and BrdU (yellow) as indicated, and merge (left) of early, mid, and late S-phase stages **(D)**. Non-replicated alleles are single spots (yellow arrows), while replicated alleles are double spots (white arrows). Number of counted nuclei was 100 under control and APH treatment (*N* = 100) for one replicate. Scale bar: 10 µm.

We wondered whether replication stress affected the dynamics of replication during the S-phase within fragile regions expressed in U-251 MG cells. To this end, we analyzed the replication timing of three CFSs (1p31.1, 3q13.3, and 7q11.2) previously studied in MRC-5 fibroblasts (Maccaroni et al., 2020) by using a specific probe for each fragile region to compare their behavior upon mild replicative stress. These replication data were also compared with the replication analysis in lymphocytes. More than 100 nuclei for each FISH probe were observed both in the absence and the presence of APH. The spots indicate the replication status of the CFS: one spot for unreplicated allele (Figure 7D, yellow arrows) and double spots for replicated alleles (Figure 7D, white arrows). Immunofluorescence (IF) against BrdU on the same nuclei revealed each specific substage of the S-phase (early, mid, and late; Figure 7D). The combined FISH-IF allowed us to monitor the CFS replication status throughout the S-phase.

In the control condition, the site 1p31.1 showed only 50% of replicated alleles in MRC-5 fibroblasts in the late S-phase; under APH treatment, we observed an increase in the total amount of replicated alleles (until ∼75%) in the late S-phase, as seen in APH-treated U-251 MG cells and lymphocytes (Figure 7A, graphs).

For 3q13.3, we did not observe any difference between untreated and treated cells in spite of being a highly expressed CFS in GBM (Figure 5F, graph). In U-251 MG cells and lymphocytes under APH stress, we observed a ∼5% reduction of replicated alleles compared to the control (Figure 7B, graphs).

Regarding the fragile site 7q11.2, in lymphocytes, it reached the same percentages of replicated alleles in the late S-phase under both normal and replicative stress conditions. Under APH, U-251 MG cells showed a slight decrease in replicated alleles, while MRC-5 fibroblasts showed a ∼10% increase in replicated alleles before the end of the S-phase (Figure 7C, graphs). However, for all the analyzed samples, we did not detect complete replications for these alleles, highlighting a possible delay in replication after the late S-phase/G2. Collectively, our data show no significant difference in replication dynamics within these regions, whether they were non-fragile (lymphocytes) or expressed as CFSs at different frequencies (U-251 MG and MRC-5 fibroblasts).

### Replication in Interphase and Mitotic Cells

**FIGURE 8:**
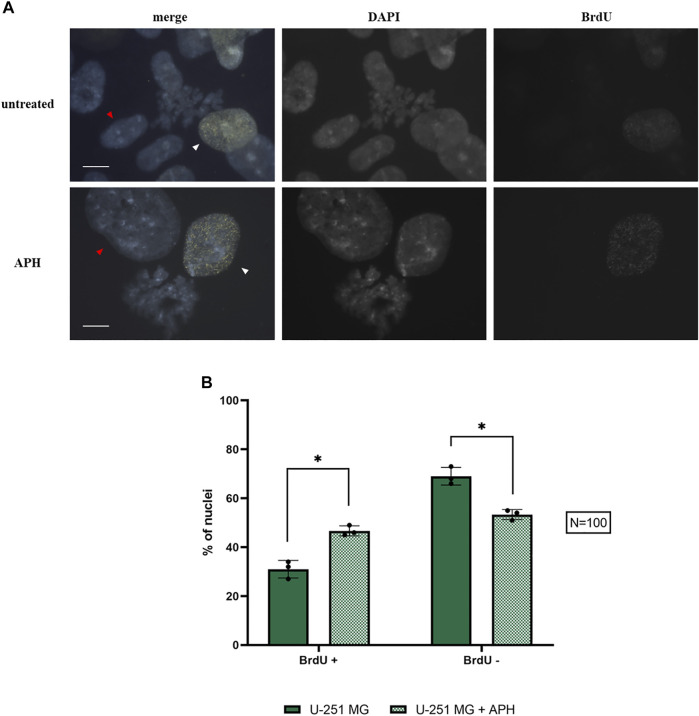
Replicating nuclei under control and APH-treated conditions with BrdU foci (yellow) and DAPI staining (blue) **(A)**; the graph indicates the amount of BrdU-negative (non-replicating cells, red arrows) and BrdU-positive (replicating cells, white arrows) glioblastoma cells under control and APH-treated conditions **(B)**. Scale bar: 10 µm. The error bars represent standard deviation (SD) determined from 3 independent experiments (*N* = 100 nuclei for each replicate). Paired *t*-test was used to calculate the *p* values, where *p* > 0.05 ns, *p ≤ 0.05, **p < 0.01, ***p < 0.001, and ****p < 0.0001.

Our data suggest that upon exposure to a low dose of replication stress, GBM cells suffer insults that result in a lower level of mitotic cells (Figure 1) and chromosome instability (Figure 2 and Figure 4), including expression of specific CFSs (Figure 5). Some of these CFSs fail to duplicate within the timeframe of the S-phase (Figure 7), potentially entering mitosis unreplicated. To better understand the nature of these CFSs’ expression, we assessed the cell cycle distribution using 5-Bromo-2′-deoxyuridine (BrdU) incorporation under untreated and APH conditions in U-251 MG cells. We scored either replicating cells (BrdU-positive nuclei, white arrows in Figure 8A and Supplementary Figure S3) representing different stages of the S-phase (as in Figure 7D) or non-replicating cells (BrdU-negative, red arrows in Figure 8A), likely representing G1 or G2.

We found a shift of glioblastoma cells exposed to replication stress from G1/G2 BrdU negative to a replicative state with a significant increase in BrdU-positive cells in APH-treated cells (Figure 8B, graph). The higher proportion of cells residing in the S-phase may imply the replication struggle induced by APH and activation of the intra-S-phase checkpoint and justify the decreased number of cells that make it into mitosis (Figure 1). We hypothesized that unfinished replication may cause MiDAS in U-251 MG cells, a phenomenon where DNA synthesis continues into mitosis. Thus, we assessed persistent BrdU incorporation on metaphase spreads (Supplementary Figure S4). Indeed, we observed the presence of BrdU signals on 1–4 chromosomes per metaphase spread only under the APH condition (Supplementary Figure S4), indicating that defective replication in U-251 MG cells is not dealt with in G2 but persists into mitosis, likely contributing to the chromosome defects as observed in Figures 2 and Figure 3 and resulting in gaps and breaks at specific CFSs (Figures 4 and Figure 5), given the small number of BrdU-positive loci seen in U-251 MG cells only after APH treatment.

**FIGURE 9:**
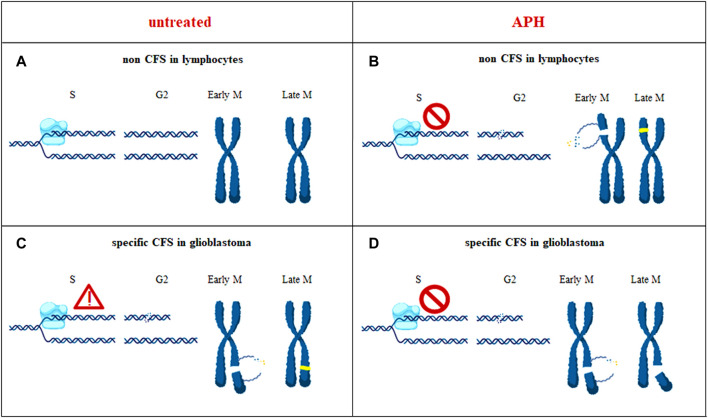
In untransformed cells such as lymphocytes, during unperturbed cell cycle progression, a chromosome region that does not show replication issue is depicted (non CFS) **(A)**. In glioblastoma cells, the same chromosomal region can have an impairment that can be rescued (CFS) **(C)**. Under an APH-induced stressful condition, glioblastoma-CFS is unable to rescue replication impairment, hereby depicted as replication fork stalling **(D)**, contrary to non CFS in lymphocytes where the impairment is overcome **(B)**; yellow dots are the new incorporated nucleotides during the repair processes. When the CFS burden is not dealt with, the site is expressed as a chromosome break **(D)**.

Collectively, our data suggest that these chromosomal regions show glioblastoma-specific CFSs likely due to multiple converging features including presence of long genes actively transcribed in the tissue of origin, slower/impaired replication, and evidence of MiDAS to attempt completing DNA synthesis at these regions ahead of chromosome segregation in mitosis (Figure 9).

## Discussion

Our work presents a characterization of CFS expression in a cell line derived from the malignant brain tumor GBM. Here, we assessed the expression of every CFS found in the human genome in U-251 MG cells under conditions of mild replicative stress using APH. Out of all known CFSs, we identified 52 CFSs expressed as gaps/breaks only after APH treatment in GBM cells and 17 CFSs that showed fragility with frequency equal to at least 1% on the total gaps/breaks. We also identified two CFSs that appear to be glioblastoma-specific localized to regions FRA2E (2p13/p12) and FRA2F (2q22), only seen as breaks in U-251 MG cells after APH treatment. Within the CFSs highly expressed in GBM after APH treatment, we observed presence of long genes, incomplete replication, and delayed DNA synthesis that persisted into mitosis (MiDAS). Given our data showing similar replication dynamics during the S-phase for untreated U-251 MG or treated with APH, we suggest that the replication issues at these sites partly activate a replication checkpoint as we observed upon scoring BrdU-positive and -negative cells. Likely, however, several cells continue into mitosis with under-replicated DNA. Presence of long genes, especially expressed in brain tissue, may further enhance the fragility of a specific region that manifests as gaps or breaks within mitotic chromosomes. We also noted fragile and uncondensed chromatin and other chromosome phenotypes, indicating that issues generated by replication stressors such as a low dose of APH result in a variety of phenotypes, including but not limited to fragile site expression that can readily compromise overall genome stability.

In cancer, DNA synthesis may be compromised by the lack of basic replication-components, leading to mitotic arrest (Miron et al., 2015). After APH treatment, we observed a specific pattern of CFSs in glioblastoma cells that is not detectable in lymphocytes and fibroblasts. This different response of the three cell types can be explained by the fact that cancer cells as glioblastoma could bypass DNA damage and the cell cycle checkpoint, including intra-S-phase ones, and proceed through the cell cycle despite the persistent damage and/or unfinished replication within the timeframe of the S-phase or G2, with the subsequent effect of aberrant mitoses and the potential for rearrangements and accumulation of chromosome aberrations.

Indeed, it can be noted that glioblastoma cells express several more CFSs than lymphocytes, implying that CFSs in primary cells such as lymphocytes may express breaks in specific regions after mild replication stress due to intrinsic and tissue-specific features; in glioblastoma, on the other hand, to these intrinsic features get added additional factors likely related to malignant transformation and decreased proficiency of checkpoints and DDR. This is in line with the notion that glioblastoma often displays high CIN. Some of the CFSs we have identified have been specifically characterized in fibroblasts (i.e., 1p31, 3q13, and 7q11; refer to Murano et al., 1989; Le Tallec et al., 2011; Maccaroni et al., 2020), suggesting that the chromosome fragility phenotype of GBM appears under our experimental conditions to be more similar to fibroblasts than lymphocytes.

Our work offers an initial overview of common fragile site expression in glioblastoma that may be further exploited for future cytogenetic, molecular, and clinical studies to advance our understanding of this incurable cancer.

## Data Availability

The original contributions presented in the study are included in the article/Supplementary Material; further inquiries can be directed to the corresponding authors.
