# Supplementary material for: Characterization of Chromosomal Instability in Glioblastoma
Source: Front Genet. 2022 Jan 28;12:810793. doi: 10.3389/fgene.2021.810793 (PMC8831864; doi:10.3389/fgene.2021.810793)
Supplement: Supplementary file 1 [file DataSheet1.PDF]

Supplementary Material

Characterization of chromosomal instability in glioblastoma

Elisa Balzano[1,2], Elena Di Tommaso[1,2], Antonio Antoccia[3], Franca Pelliccia[1*] and Simona Giunta[2*]

[1]Laboratory of Molecular Cytogenetics, Sapienza Università di Roma, Dipartimento di Biologia e Biotecnologie "Charles Darwin", Roma 00185, Italy

[2]Laboratory of Genome Evolution, Sapienza Università di Roma, Dipartimento di Biologia e Biotecnologie "Charles Darwin", Roma 00185, Italy

[3]Laboratory of Genetics and Cytogenetics, Università Degli Studi Roma Tre, Dipartimento di Scienze, Roma 00146, Italy

*** Correspondence:**
Franca Pelliccia
franca.pelliccia@uniroma1.it

Simona Giunta
simona.giunta@uniroma1.it

Content:

- Supplementary Figures 1-4
- Supplementary Table 1

Supplementary Figure 1. Percentage of nuclei aberrations: Blebbing (A, black arrows), cytoplasmic bridges (B, red arrow) and micronuclei (C, white arrow). We observed no significant change between untreated and APH treated cells for each nuclear aberration category. Scale bar: 10 µm. The error bars represent standard deviation (SD) determined from 3 independent experiments (N=500 nuclei for each replicate). Paired *t* test for *p* values $p>0.05$ ns (not significant), $^{*}p\leq0.05$, $^{**}p<0.01$, $^{***}p<0.001$, and $^{****}p<0.0001$.

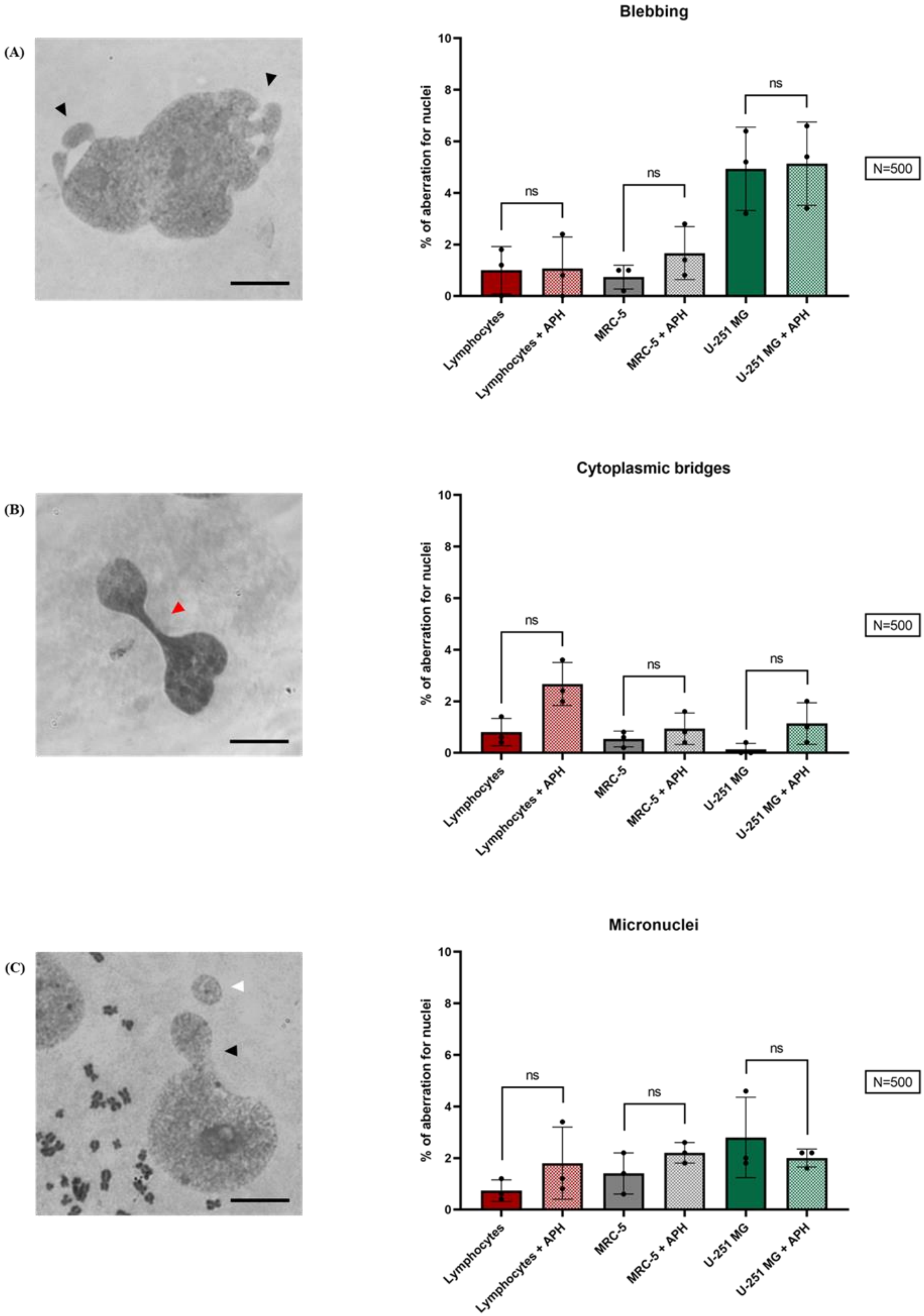
(A)
Blebbing
% of aberration for nuclei
ns
N=500
Lymphocytes
Lymphocytes + APH
MRC-5
MRC-5 + APH
U-251 MG
U-251 MG + APH
(B)
Cytoplasmic bridges
% of aberration for nuclei
ns
N=500
Lymphocytes
Lymphocytes + APH
MRC-5
MRC-5 + APH
U-251 MG
U-251 MG + APH
(C)
Micronuclei
% of aberration for nuclei
ns
N=500
Lymphocytes
Lymphocytes + APH
MRC-5
MRC-5 + APH
U-251 MG
U-251 MG + APH

Supplementary Figure 2. Schematic representation of the most expressed Fragile Sites in glioblastoma cells: 1p31.1 (A), 3q13.3 (B) and 7q11.1 (C). In each ideogram, we indicated only the most expressed genes in the brain. Colors correspond to different genes localized in these specific regions (delimitated by the red brackets). Highlighted genes in blue brackets are the antisense RNAs (asRNAs) and intronic RNAs (itRNAs).

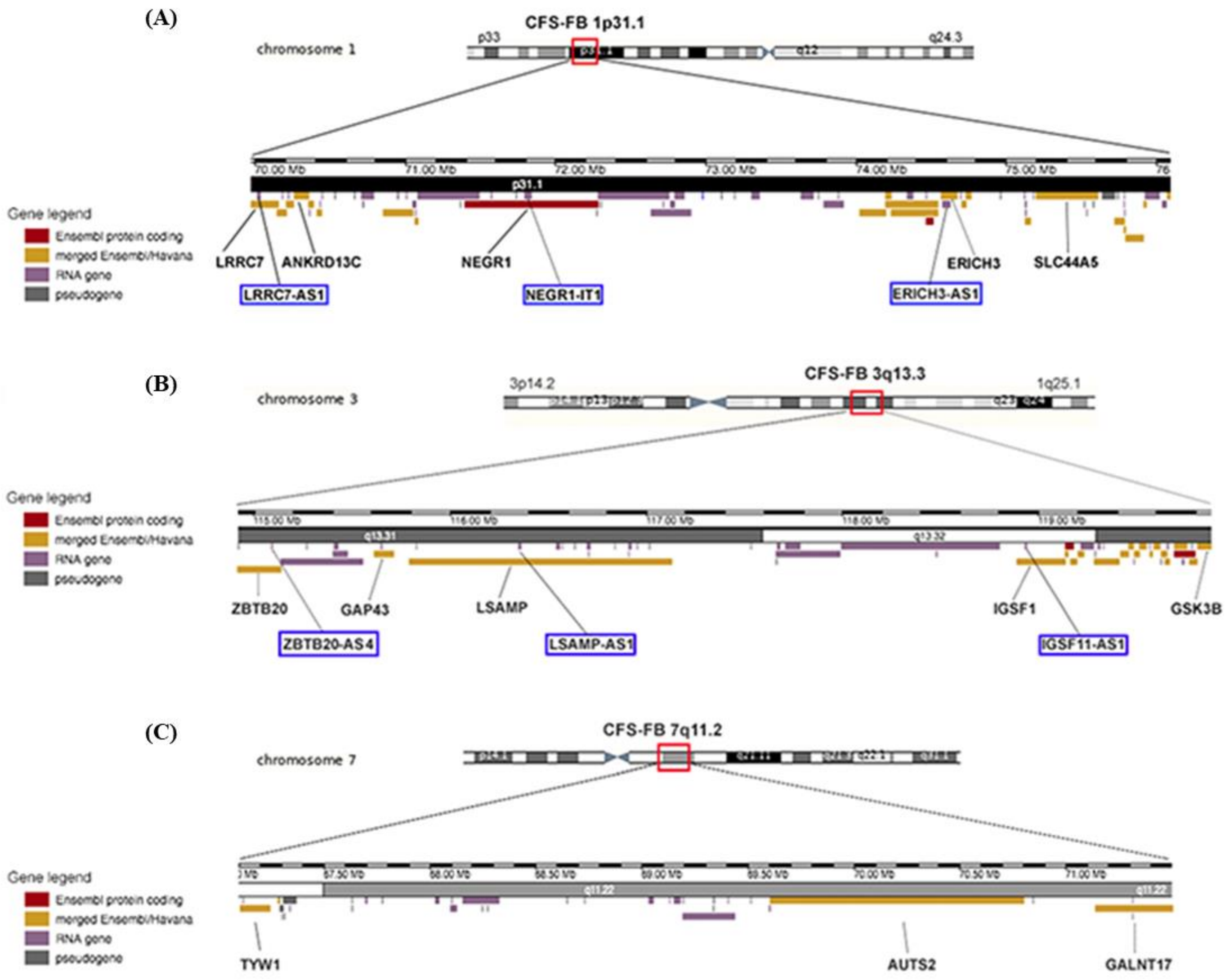

Supplementary Figure 3. Replicating nuclei with BrdU foci (yellow) and DAPI staining (blue); E: early S-phase, M: mid S-phase, L: late S-phase. Scale bar: 10 µm.

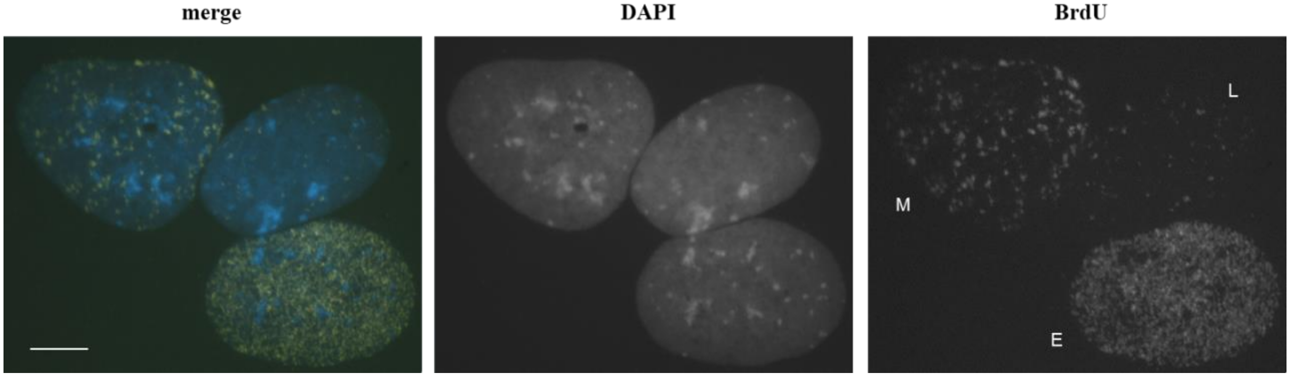

Supplementary Figure 4. U-251 MG metaphases in control and APH treated conditions. Chromosomes were observed by DAPI staining (blue) and BrdU foci (yellow). BrdU foci colocalizing with mitotic chromosomes, highlighted by brackets, were observed only in APH-treated metaphase spreads. Scale bar: 10 µm.

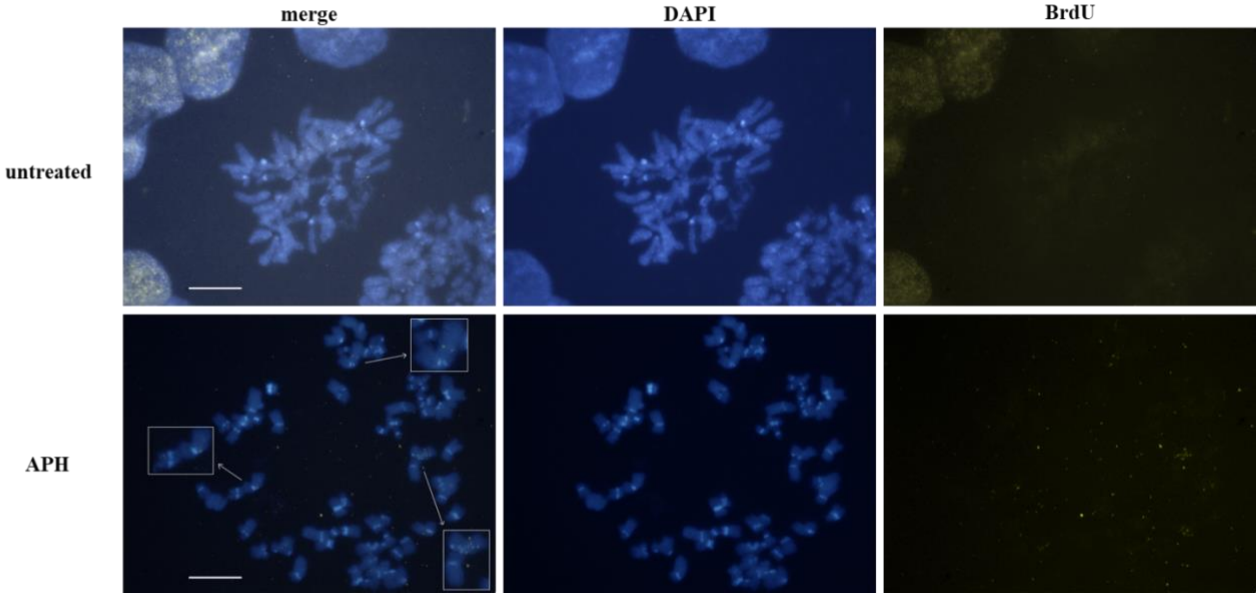

Supplementary Table 1. Left column: Genomic location of the five CFSs peculiarly expressed in U-251 MG cells. Right column: Brain-expressed genes found and mapped within these CFSs. All locations, length and gene expression in the brain derived from three International Human Genome Scientific Resources: NCBI: https://www.ncbi.nlm.nih.gov/genome/gdv/ (Release Data May 16, 2021); Ensembl: http://www.ensembl.org/Homo_sapiens/Location/Genome (Human GRCh38p13); GeneCards: https://www.genecards.org/

**Genes Expressed in Brain**	**1p31.1 (70,800,009 - 76,000,000 = ~ 5,2 Mb)**
**Location:**<br>69,567,922-<br>70,144,364<br>forward strand	**LRRC7, leucine rich repeat containing 7: (576,443 nt)**<br>**Biased expression in brain (RPKM 2.3 ± 1,46),** testis (RPKM 0.6 ± 0,23) and 2 other tissues.<br>**GeneCards:** LRRC7 (Leucine Rich Repeat Containing 7) is a Protein Coding gene. Diseases associated with LRRC7 include Cri-Du-Chat Syndrome and Chromosome 3Q29 Deletion Syndrome. Among its related pathways are Innate Immune System.<br>**Prot-Summary**: Required for normal synaptic spine architecture and function. Necessary for DISC1 and GRM5 localization to postsynaptic density complexes and for both N-methyl D-aspartate receptor-dependent and metabotropic glutamate receptor-dependent long term depression.
**Location:**<br>70,020,823-<br>70,042,261<br>reverse strand	**LRRC7-AS1, LRRC7 Antisense RNA 1 (lncRNA): (21,439 nt)**<br>**Biased expression in brain (RPKM 1,1 ± 0,59),** testis (RPKM 1,2.8) and 3 other tissues.
**Location:**<br>70,258,999-<br>70,354,734<br>reverse strand	**ANKRD13C ankyrin repeat domain 13C: (95,979 nt)**<br>**Ubiquitous expression in brain (RPKM 5.7 ± 0,98)**, endometrium (RPKM 4.8) and 25 other tissues.<br>**GeneCards:** is a Protein Coding gene. Gene Ontology (GO) annotations related to this gene include *signaling receptor binding*.<br>**Prot-Summary**: Acts as a molecular chaperone for G protein-coupled receptors, regulating their biogenesis and exit from the ER.
**Location:**<br>71,395,943-<br>72,282,539<br>reverse strand	**NEGR1, neuronal growth regulator 1: (886,597 nt)**<br>**Broad expression in brain (RPKM 13.7 ± 5,8)**, fat (RPKM 5.3) and 18 other tissues.<br>**GeneCards:** is a Protein Coding gene. Diseases associated with NEGR1 include Nieman-Pick and Leptin Deficiency Or Dysfunction. Among its related pathways are Metabolism of proteins and Cell adhesion molecules. An important paralog of this gene is LSAMP (FB 3q13.3).<br>**Prot-Summary**: May be involved in cell-adhesion. May function as a trans-neural growth-promoting factor in regenerative axon sprouting in the mammalian brain (By similarity).
**Location:**<br>71,794,232-<br>71,837,012<br>reverse strand	**NEGR1-IT1, Intronic Transcript 1 (RNA Gene): (42,781 nt)**
**Location:**	**ERICH3, glutamate rich 3: (106,231 nt)**

74,568,113-<br>74,674,343<br>reverse strand	Biased expression in testis (RPKM 11.4), **brain (RPKM 6.8 ±3,46)** and 2 other tissues.
**Location:**<br>74,577,430-<br>74,626,098<br>forward strand	**ERICH3-AS1, ERICH3 Antisense RNA 1: (48,669 nt)**<br>Biased expression in testis (RPKM 1.5), **brain (RPKM 0.5 ±0,26)** and 2 other tissues.
**Location:**<br>75,202,129-<br>75,724,261<br>reverse strand	**SLC44A5, solute carrier family 44 member 5: (522,133 nt)**<br>Broad expression in skin (RPKM 2.6), testis (RPKM 1.3), **brain (RPKM 1,18 ± 0,33)** and 20 other tissues.<br>**GeneCards:** is a Protein Coding gene. Diseases associated with SLC44A5 include Choline Deficiency Disease. Among its related pathways are Glycerophospholipid biosynthesis and Transport of glucose and other sugars, bile salts and organic acids, metal ions and amine compounds.
**Genes Expressed in Brain**	**FRA2E (2p13.2-p12.1) (71,100,000 - 80,100,000 = ~ 9,0 Mb)**
**Location:**<br>72,175,984-<br>72,826,041<br>reverse strand	**EXOC6B, exocyst complex component 6B: (650,050 nt)**<br>Ubiquitous expression in heart (RPKM 12.8+- 2,7), **brain (RPKM 8.7 ±0,34)** and 23 other tissues.<br>This gene encodes a protein which is a part of the evolutionarily conserved exocyst, a multimeric protein complex necessary for exocytosis, which in turn, is crucial for cell growth, polarity and migration. Disruption of this gene may be associated with phenotypes exhibiting multiple symptoms including intellectual disability and developmental delay (DD).<br>**GeneCards:** Diseases associated with EXOC6B include Spondyloepimetaphyseal Dysplasia With Joint Laxity, Type 3 and Spondyloepimetaphyseal Dysplasia With Joint Laxity. Among its related pathways are Cytoskeleton remodeling_RalA regulation pathway.<br>**Prot-Summary:** Component of the exocyst complex involved in the docking of exocytic vesicles with fusion sites on the plasma membrane.
**Location:**<br>72,175,984-<br>72,826,041<br>reverse strand	**SFXN5, sideroflexin 5: (129,677 nt)**<br>**Broad expression in brain (RPKM 13.0 ± 4,6)**, liver (RPKM 3.8) and 20 other tissues.<br>**GeneCards:** Diseases associated with SFXN5 include Parkinson Disease, Late-Onset. Gene Ontology (GO) annotations related to this gene include *cation transmembrane transporter activity* and *citrate transmembrane transporter activity*.<br>**Prot-Summary**: Mitochondrial amino-acid transporter (By similarity). Does not act as a serine transporter: not able to mediate transport of serine into mitochondria (PubMed:30442778).
**Location:**<br>73,385,758-<br>73,609,919<br>forward strand	**ALMS1, centrosome and basal body associated protein: (224,162 nt)** Broad expression in testis (RPKM 15.5), ovary (RPKM 5.0), **brain (RPKM 1,39 ± 0,34)** and 20 other tissues.<br>This gene encodes a protein containing a large tandem-repeat domain as well as additional low complexity regions. The encoded protein functions in microtubule

	organization, particularly in the formation and maintenance of cilia. Mutations in this gene cause Alstrom syndrome.<br>**GeneCards:** Diseases associated with ALMS1 include Alstrom Syndrome and Stargardt Disease 1. Among its related pathways are Cell Cycle, Mitotic and Regulation of PLK1 Activity at G2/M Transition.<br>**Prot-Summary**: Involved in PCM1-dependent intracellular transport. Required, directly or indirectly, for the localization of NCAPD2 to the proximal ends of centrioles. Required for proper formation and/or maintenance of primary cilia (PC), microtubule-based structures that protrude from the surface of epithelial cells.
**Location:**<br>73,456,764-<br>73,459,482<br>forward strand	**ALMS1-IT1, ALMS1 intronic transcript 1: (2,718 nt)**
**Location:**<br>73,469,525-<br>73,471,030<br>forward strand	**novel transcript, sense intronic to ALMS1: (1,778 nt)**
**Location:**<br>73,828,916-<br>73,873,659<br>forward strand	**STAMBP, STAM binding protein: (44,746 nt)**<br>Ubiquitous expression in thyroid (RPKM 4.9), **brain (RPKM 4.5 ± 1,26)** and 25 other tissues.<br>Cytokine-mediated signal transduction in the JAK-STAT cascade requires the involvement of adaptor molecules. One such signal-transducing adaptor molecule contains an SH3 domain that is required for induction of MYC and cell growth. The protein encoded by this gene binds to the SH3 domain of the signal-transducing adaptor molecule, and plays a critical role in cytokine-mediated signaling for MYC induction and cell cycle progression. Multiple alternatively spliced transcript variants encoding the same protein isoform have been found for this gene.<br>**GeneCards:** Diseases associated with STAMBP include Microcephaly-Capillary Malformation Syndrome and Camptodactyly-Arthropathy-Coxa Vara-Pericarditis Syndrome. Among its related pathways are Internalization of ErbB1 and Protein ubiquitination. Gene Ontology (GO) annotations related to this gene include *protein domain specific binding* and thiol-dependent ubiquitin-specific protease activity. An important paralog of this gene is STAMBPL1.<br>**Prot-Summary**: Zinc metalloprotease that specifically cleaves 'Lys-63'-linked polyubiquitin chains. Does not cleave 'Lys-48'-linked polyubiquitin chains (By similarity). Plays a role in signal transduction for cell growth and MYC induction mediated by IL-2 and GM-CSF. Potentiates BMP (bone morphogenetic protein) signaling by antagonizing the inhibitory action of SMAD6 and SMAD7. Has a key role in regulation of cell surface receptor-mediated endocytosis and ubiquitin-dependent sorting of receptors to lysosomes. Endosomal localization of STAMBP is required for efficient EGFR degradation but not for its internalization (By similarity). Involved in the negative regulation of PI3K-AKT-mTOR and RAS-MAP signaling pathways.

**Location:**<br>73,982,036-<br>74,135,394<br>forward strand	**TET3, tet methylcytosine dioxygenase 3: (153,359 nt)**<br>Ubiquitous expression in bone marrow (RPKM 6.8), skin (RPKM 6.6), **brain (RPKM 2,94)** and 22 other tissues.<br>Members of the ten-eleven translocation (TET) gene family, including TET3, play a role in the DNA methylation process (Langemeijer et al., 2009 [PubMed 19923888])<br>**GeneCards:** TET3 (Tet Methylcytosine Dioxygenase 3) is a Protein Coding gene. Diseases associated with TET3 include Beck-Fahrner Syndrome and Miles-Carpenter Syndrome. Among its related pathways are Cytosine methylation and Gene Expression. Gene Ontology (GO) annotations related to this gene include *methylcytosine dioxygenase activity*.<br>**Prot-Summary**: Dioxygenase that catalyzes the conversion of the modified genomic base 5-methylcytosine (5mC) into 5-hydroxymethylcytosine (5hmC) and plays a key role in epigenetic chromatin reprogramming in the zygote following fertilization (PubMed:31928709). Also mediates subsequent conversion of 5hmC into 5-formylcytosine (5fC), and conversion of 5fC to 5-carboxylcytosine (5caC). Conversion of 5mC into 5hmC, 5fC and 5caC probably constitutes the first step in cytosine demethylation (By similarity). Selectively binds to the promoter region of target genes and contributes to regulate the expression of numerous developmental genes (PubMed:23217707). In zygotes, DNA demethylation occurs selectively in the paternal pronucleus before the first cell division, while the adjacent maternal pronucleus and certain paternally-imprinted loci are protected from this process. Participates in DNA demethylation in the paternal pronucleus by mediating conversion of 5mC into 5hmC, 5fC and 5caC. Does not mediate DNA demethylation of maternal pronucleus because of the presence of DPPA3/PGC7 on maternal chromatin that prevents TET3-binding to chromatin (By similarity). In addition to its role in DNA demethylation, also involved in the recruitment of the O-GlcNAc transferase OGT to CpG-rich transcription start sites of active genes, thereby promoting histone H2B GlcNAcylation by OGT (PubMed:23353889). Binds preferentially to DNA containing cytidine-phosphate-guanosine (CpG) dinucleotides over CpH (H=A, T, and C), hemimethylated-CpG and hemimethylated-hydroxymethyl-CpG (PubMed:29276034).
**Location:**<br>75,046,463-<br>75,199,520<br>reverse strand	**TACR1, tachykinin receptor 1: (153,058 nt)**<br>Broad expression in skin (RPKM 1.8), endometrium (RPKM 1.4), **brain (RPKM 1,38 ± 0,97)** and 16 other tissues.<br>This gene belongs to a gene family of tachykinin receptors. These tachykinin receptors are characterized by interactions with G proteins and contain seven hydrophobic transmembrane regions. This gene encodes the receptor for the tachykinin substance P, also referred to as neurokinin 1. The encoded protein is also involved in the mediation of phosphatidylinositol metabolism of substance P.<br>**GeneCards:** TACR1 (Tachykinin Receptor 1) is a Protein Coding gene. Diseases associated with TACR1 include Cystitis and Causalgia. Among its related pathways are Neuropathic Pain-Signaling in Dorsal Horn Neurons and Clathrin-mediated endocytosis. Gene Ontology (GO) annotations related to this gene include *G protein-coupled receptor activity* and *substance P receptor activity*.

	**Prot-Summary**: This is a receptor for the tachykinin neuropeptide substance P. It is probably associated with G proteins that activate a phosphatidylinositol-calcium second messenger system. The rank order of affinity of this receptor to tachykinins is: substance P > substance K > neuromedin-K.
**Location: 76,747,685-77,593,319** reverse strand	**LRRTM4, leucine rich repeat transmembrane neuronal 4: (774,692 nt)**<br>**Biased expression in brain (RPKM 5.6 ±1,098)** and lung (RPKM 0.2).<br>**GeneCards:** Diseases associated with LRRTM4 include Schizophrenia 4 and Schizophrenia 18.<br>**Prot-Summary**: May play a role in the development and maintenance of the vertebrate nervous system. Exhibits strong synaptogenic activity, restricted to excitatory presynaptic differentiation
**Location: 79,505,054-80,648,788** forward strand	**CTNNA2, catenin alpha 2: (1,143,735 nt)**<br>**Biased expression in brain (RPKM 26.7 ±12,117)**, testis (RPKM 6.2) and 1 other tissue.<br>**GeneCards:** Diseases associated with CTNNA2 include Cortical Dysplasia, Complex, With Other Brain Malformations 9 and Hereditary Breast Ovarian Cancer Syndrome. Among its related pathways are Adherens junction and Adhesion.<br>**Prot-Summary:** May function as a linker between cadherin adhesion receptors and the cytoskeleton to regulate cell-cell adhesion and differentiation in the nervous system. Required for proper regulation of cortical neuronal migration and neurite growth. It acts as negative regulator of Arp2/3 complex activity and Arp2/3-mediated actin polymerization. It thereby suppresses excessive actin branching which would impair neurite growth and stability. Regulates morphological plasticity of synapses and cerebellar and hippocampal lamination during development. Functions in the control of startle modulation.
	**FRA2F (2q21.3-q22) (133,100,000-141,100,000 = ~8,0 Mb)**
**Location: 132,671,788-133,675,182** reverse strand	**NCKAP5, NCK associated protein 5: (1,003,395 nt)**<br>Broad expression in lung (RPKM 2.7+- 0,6, kidney (RPKM 0.7 +- 0,2), **brain (0,6 ± 0,14)** and 19 other tissues.<br>**GeneCards:** Diseases associated with NCKAP5 include Attention Deficit-Hyperactivity Disorder.<br>**Prot-Summary: No data available**
**Location: 134,119,922-134,454,621** forward strand	**MGAT5, MGAT5 alpha-1,6-mannosylglycoprotein 6-beta-N-acetylglucosaminyltransferase: (334,700 nt)**<br>Ubiquitous expression in heart (RPKM 14.6), lymph node (RPKM 12.4), **brain (RPKM 11.216 ±1,51)** and 23 other tissues.<br>The protein encoded by this gene belongs to the glycosyltransferase family. It catalyzes the addition of beta-1,6-N-acetylglucosamine to the alpha-linked mannose of biantennary N-linked oligosaccharides present on the newly synthesized glycoproteins. It is one of the most important enzymes involved in the regulation of the biosynthesis of glycoprotein oligosaccharides. Alterations of the oligosaccharides on cell surface glycoproteins cause significant changes in the adhesive or migratory behavior of a cell. Increase in the activity of this enzyme has been correlated with the progression of invasive malignancies.

	**GeneCards:** Diseases associated with MGAT5 include Hepatocellular Carcinoma and Colorectal Cancer. Among its related pathways are N-glycan antennae elongation in the medial/trans-Golgi and Metabolism of proteins. Gene Ontology (GO) annotations related to this gene include *acetylglucosaminyltransferase activity* and *alpha-1,6-mannosylglycoprotein 6-beta-N-acetylglucosaminyltransferase activity*.<br>**Prot-Summary:** Catalyzes the addition of N-acetylglucosamine (GlcNAc) in beta 1-6 linkage to the alpha-linked mannose of biantennary N-linked oligosaccharides (PubMed:10395745, PubMed:30140003). Catalyzes an important step in the biosynthesis of branched, complex-type N-glycans, such as those found on EGFR, TGFR (TGF-beta receptor) and CDH2 (PubMed:10395745, PubMed:22614033, PubMed:30140003). Via its role in the biosynthesis of complex N-glycans, plays an important role in the activation of cellular signaling pathways, reorganization of the actin cytoskeleton, cell-cell adhesion and cell migration. MGAT5-dependent EGFR N-glycosylation enhances the interaction between EGFR and LGALS3 and thereby prevents rapid EGFR endocytosis and prolongs EGFR signaling. Required for efficient interaction between TGFB1 and its receptor. Enhances activation of intracellular signaling pathways by several types of growth factors, including FGF2, PDGF, IGF, TGFB1 and EGF. MGAT5-dependent CDH2 N-glycosylation inhibits CDH2-mediated homotypic cell-cell adhesion and contributes to the regulation of downstream signaling pathways. Promotes cell migration. Contributes to the regulation of the inflammatory response. MGAT5-dependent TCR N-glycosylation enhances the interaction between TCR and LGALS3, limits agonist-induced TCR clustering, and thereby dampens TCR-mediated responses to antigens. Required for normal leukocyte evasation and accumulation at sites of inflammation (By similarity). Inhibits attachment of monocytes to the vascular endothelium and subsequent monocyte diapedesis (PubMed:22614033).
**Location: 134,455,759-134,719,000** forward strand	**TMEM163, transmembrane protein 163: (265,176 nt)**<br>Broad expression in lung (RPKM 3.46), **brain (RPKM 3.11 ±0,553)** and 15 other tissues.<br>**GeneCards:** Diseases associated with TMEM163 include Febrile Seizures, Familial, 7 and Febrile Seizures, Familial, 2.<br>**Prot Summary:** May bind zinc and other divalent cations and recruit them to vesicular organelles.
**Location: 135,052,292-135,176,394** forward strand	**RAB3GAP1, RAB3 GTPase activating protein catalytic subunit 1: (124,387 nt)**<br>**Ubiquitous expression in brain (RPKM 16.8 ±3.52),** thyroid (RPKM 15.9) and 25 other tissues).<br>This gene encodes the catalytic subunit of a Rab GTPase activating protein. The encoded protein forms a heterodimer with a non-catalytic subunit to specifically regulate the activity of members of the Rab3 subfamily of small G proteins. This protein mediates the hydrolysis of GTP bound Rab3 to the GDP bound form. Mutations in this gene are associated with Warburg micro syndrome 1 and Martsolf Syndrome 2. Alternate splicing results in multiple transcript variants.<br>**GeneCards:** Diseases associated with RAB3GAP1 include Warburg Micro Syndrome 1 and Martsolf Syndrome 2. Among its related pathways are Vesicle-mediated transport and COPI-independent Golgi-to-ER retrograde traffic. Gene

	Ontology (GO) annotations related to this gene include *GTPase activator activity* and *Rab guanyl-nucleotide exchange factor activity*.<br>**Prot-Summary:** Probable catalytic subunit of a GTPase activating protein that has specificity for Rab3 subfamily (RAB3A, RAB3B, RAB3C and RAB3D). Rab3 proteins are involved in regulated exocytosis of neurotransmitters and hormones. Specifically converts active Rab3-GTP to the inactive form Rab3-GDP. Required for normal eye and brain development. May participate in neurodevelopmental processes such as proliferation, migration and differentiation before synapse formation, and non-synaptic vesicular release of neurotransmitters
**Location: 135,531,455-135,725,270** forward strand	**R3HDM1, R3H domain containing 1: (193,786 nt)**<br>**Broad expression in brain (RPKM 20.1 ±6,77)**, testis (RPKM 10.5) and 23 other tissues.<br>**GeneCards:** Diseases associated with R3HDM1 include Ichthyosis, Congenital, Autosomal Recessive 11 and Autosomal Recessive Congenital Ichthyosis. Gene Ontology (GO) annotations related to this gene include *nucleic acid binding*.<br>**Prot-Summary:** No data available
**Location: 137,964,068-138,016,364** forward strand	**HNMT, histamine N-methyltransferase: (52,297 nt)**<br>Ubiquitous expression in liver (RPKM 20.0), fat (RPKM 16.4), **brain (RPKM 7,08 ± 3,4)** and 23 other tissues.<br>In mammals, histamine is metabolized by two major pathways: N(tau)-methylation via histamine N-methyltransferase and oxidative deamination via diamine oxidase. This gene encodes the first enzyme which is found in the cytosol and uses S-adenosyl-L-methionine as the methyl donor. In the mammalian brain, the neurotransmitter activity of histamine is controlled by N(tau)-methylation as diamine oxidase is not found in the central nervous system. A common genetic polymorphism affects the activity levels of this gene product in red blood cells. Multiple alternatively spliced transcript variants that encode different proteins have been found for this gene.<br>**GeneCards:** Diseases associated with HNMT include Mental Retardation, Autosomal Recessive 51 and Asthma. Among its related pathways are methionine salvage cycle III and Peptide chain elongation. Gene Ontology (GO) annotations related to this gene include *N-methyltransferase activity* and *histamine N-methyltransferase activity*.<br>**Prot-Summary:** Inactivates histamine by N-methylation. Plays an important role in degrading histamine and in regulating the airway response to histamine.
**Location: 138,669,157-138,780,390** reverse strand	**NXPH2, neurexophilin 2: (111,234 nt)**<br>Biased expression in ovary (RPKM 3.7), kidney (RPKM 2.6), **brain (RPKM 2,1 ±0,867)** and 1 other tissue.<br>**GeneCards:** Diseases associated with NXPH2 include Meier-Gorlin Syndrome 2 and Epilepsy, Idiopathic Generalized 9.<br>**Prot-Summary:** May be signaling molecules that resemble neuropeptides and that act by binding to alpha-neurexins and possibly other receptors.
**Location:** 139,234,673-139,379,146 reverse strand	**LOC105373643, uncharacterized LOC105373643: (144,474 nt) ncRNA (NCBI)**<br>**Restricted expression toward brain (RPKM 1.8 ±2,466).**

**Location**<br>140,231,423-142,132,463<br>reverse strand	**LRP1B, LDL receptor related protein 1B: ((1,901,041 nt)**<br>**Biased expression in brain (RPKM 3.0 ±1,531)**, thyroid (RPKM 1.8) and 4 other tissues.<br>This gene encodes a member of the low density lipoprotein (LDL) receptor family. These receptors play a wide variety of roles in normal cell function and development due to their interactions with multiple ligands. Disruption of this gene has been reported in several types of cancer.<br>**GeneCards:** Diseases associated with LRP1B include Lung Canter and Meier-Gorlin Syndrome 2. Gene Ontology (GO) annotations related to this gene include calcium ion binding and low-density lipoprotein particle receptor activity.<br>**Prot-Summary:** Potential cell surface proteins that bind and internalize ligands in the process of receptor-mediated endocytosis.<br>**CIViC Summary for LRP1B Gene:** LRP1B is a putative tumor suppressor and a member of the low-density lipoprotein (LDL) receptor family. The LDL receptor family have roles related to clearance of extracellular ligand and are proposed to be involved in extracellular signal transduction. silencing and down-expression of LRP1B as been observed in renal cell carcinoma and thyroid cancer. Further Deletion of LRP1B has been associated with chemotherapy resistance in high-grade serous cancers.
**Genes Expressed in Brain**	**3q13.3 3q13.3 (114,900,000-119,900,000 = ~ 5,0 Mb)**
**Location:**<br>114,314,500-115,147,288<br>reverse strand	**ZBTB20, zinc finger and BTB domain containing 20: (832,789 nt)**<br>Ubiquitous expression in kidney (RPKM 4.0), ovary (RPKM 3.7), **brain (RPKM 3,15 ± 1,72)** and 24 other tissues.<br>This gene acts as a transcriptional repressor and plays a role in many processes including neurogenesis, glucose homeostasis, and postnatal growth. Mutations in this gene have been associated with Primrose syndrome as well as the 3q13.31 microdeletion syndrome. Alternative splicing results in multiple transcript variants encoding distinct isoforms.<br>**GeneCards:** Diseases associated with ZBTB20 include Primrose Syndrome and Disease Of Mental Health. Gene Ontology (GO) annotations related to this gene include *proximal promoter DNA-binding transcription repressor activity, RNA polymerase II-specific*.<br>**Prot-Summary:** May be a transcription factor that may be involved in hematopoiesis, oncogenesis, and immune responses (PubMed:11352661). Plays a role in postnatal myogenesis, may be involved in the regulation of satellite cells self-renewal (By similarity)
**Location:**<br>115,623,510-115,721,485<br>forward strand	**GAP43, growth associated protein 43: (97,976 nt)**<br>**Biased expression in brain (RPKM 55.1 ± 19,86)** and appendix (RPKM 1.7).<br>The protein encoded by this gene has been termed a 'growth' or 'plasticity' protein because it is expressed at high levels in neuronal growth cones during development and axonal regeneration. This protein is considered a crucial component of an effective regenerative response in the nervous system. Alternatively spliced transcript variants encoding distinct isoforms have been found for this gene.

	**GeneCards:** GAP43 (Growth Associated Protein 43) is a Protein Coding gene. Diseases associated with GAP43 include Developmental Coordination Disorder and Status Epilepticus. Among its related pathways are Cytoskeletal Signaling and Developmental Biology. Gene Ontology (GO) annotations related to this gene include *calmodulin binding*.<br>**Prot-Summary:** This protein is associated with nerve growth. It is a major component of the motile 'growth cones' that form the tips of elongating axons. Plays a role in axonal and dendritic filopodia induction.
**Location:**<br>115,802,374-116,445,487 (NCBI)<br><br>115,802,363-117,139,389 (ENSEMBL)<br>reverse strand	**LSAMP, limbic system associated membrane protein: (NCBI: 643,114 nt – Ensembl: 1,337,026 nt)**<br>**Biased expression in brain (RPKM 19.9 ±1,35)**, prostate (RPKM 5.4) and 9 other tissues.<br>This gene encodes a member of the immunoglobulin LAMP, OBCAM and neurotrimin (IgLON) family of proteins. The encoded preproprotein is proteolytically processed to generate a neuronal surface glycoprotein. This protein may act as a selective homophilic adhesion molecule during axon guidance and neuronal growth in the developing limbic system. The encoded protein may also function as a tumor suppressor and may play a role in neuropsychiatric disorders. Alternative splicing results in multiple transcript variants, at least one of which encodes a preproprotein that is proteolytically processed.<br>**GeneCards:** Diseases associated with LSAMP include Ring Dermoid Of Cornea and Chromosome 1Q21.1 Duplication Syndrome. Among its related pathways are Metabolismi of proteins and Post-translational modification- synthesis of GPI-anchored proteins. An important paralog of this gene is NEGR1 (FB 1p31.1).<br>**Prot-Summary:** Mediates selective neuronal growth and axon targeting. Contributes to the guidance of developing axons and remodeling of mature circuits in the limbic system. Essential for normal growth of the hyppocampal mossy fiber projection (By similarity)
**Location:**<br>116,360,024-116,370,085<br>forward strand	**LSAMP-AS1, LSAMP antisense RNA 1: (10,062 nt)**
**Location:**<br>118,900,557-119,146,064<br>reverse strand	**IGSF11, immunoglobulin superfamily member 11: (245,508 nt)**<br>Biased expression in testis (RPKM 12.0**), brain (RPKM 8.3 ±3,23)** and 4 other tissues.<br>IGSF11 is an immunoglobulin (Ig) superfamily member that is preferentially expressed in brain and testis. It shares significant homology with coxsackievirus and adenovirus receptor (CXADR; MIM 602621) and endothelial cell-selective adhesion molecule (ESAM).<br>**GeneCards:** Diseases associated with IGSF11 include Lymphogranuloma Venereum. Among its related pathways are Cell adhesion molecules.<br>**Prot-Summary:** Functions as a cell adhesion molecule through homophilic interaction. Stimulates cell growth.
**Location:**	**IGSF11-AS1, antisense RNA 1: (5,166 nt)**

118,943,076-<br>118,948,241<br>forward strand	
**Genes Expressed in Brain**	**7q11.2 7q11.2 (67,700,000 - 71,400,000 = ~ 3,7 Mb)**
**Location:**<br>66,996,830-<br>67,239,515<br>forward strand	**TYW1, tRNA-yW synthesizing protein 1 homolog: (242,686 nt)**<br>Ubiquitous expression in thyroid (RPKM 5.7), ovary (RPKM 5.0), **brain (RPKM ±2.28)** and 24 other tissues.<br>Wybutosine (yW) is a hypermodified guanosine found in phenylalanine tRNA adjacent to the anticodon that stabilizes codon-anticodon interactions in the ribosome. In yeast, the homolog of this gene is essential for the synthesis of wybutosine. Alternative splicing results in multiple transcript variants.<br>**GeneCards:** TYW1 (TRNA-YW Synthesizing Protein 1 Homolog) is a Protein Coding gene. Diseases associated with TYW1 include Sorsby Fundus Dystrophy. Among its related pathways are tRNA processing and Gene Expression. Gene Ontology (GO) annotations related to this gene include *oxidoreductase activity* and *4 iron, 4 sulfur cluster binding*.<br>**Prot-Summary:** Wybutosine is a hyper modified guanosine with a tricyclic base found at the 3'-position adjacent to the anticodon of eukaryotic phenylalanine tRNA. Catalyzes the condensation of N-methylguanine with 2 carbon atoms from pyruvate to form the tricyclic 4-demethylwyosine, an intermediate in wybutosine biosynthesis (By similarity)
**Location:**<br>69,598,475-<br>70,793,506<br>forward strand	AUTS2, activator of transcription and developmental regulator AUTS2: **(1,195,032 nt)**<br><br>Ubiquitous expression in salivary gland (RPKM 2.5), skin (RPKM 2.4), **brain 1,93 ±0,21** and 25 other tissues.<br>This gene has been implicated in neurodevelopment and as a candidate gene for numerous neurological disorders, including autism spectrum disorders, intellectual disability, and developmental delay. Mutations in this gene have also been associated with non-neurological disorders, such as acute lymphoblastic leukemia, aging of the skin, early-onset androgenetic alopecia, and certain cancers. Alternative splicing results in multiple transcript variants encoding different isoforms.<br>Ubiquitous expression in salivary gland (RPKM 2.5), skin (RPKM 2.4) and 25 other tissues<br>**GeneCards:** AUTS2 (Activator Of Transcription And Developmental Regulator AUTS2) is a Protein Coding gene. Diseases associated with AUTS2 include Mental Retardation, Autosomal Dominant 26 and Multiple Congenital Anomalies/Dysmorphic Syndrome-Intellectual Disability. An important paralog of this gene is FBRSL1.<br>**Prot-Summary:** Component of a Polycomb group (PcG) multiprotein PRC1-like complex, a complex class required to maintain the transcriptionally repressive state of many genes, including Hox genes, throughout development. PcG PRC1 complex acts via chromatin remodeling and modification of histones; it mediates monoubiquitination of histone H2A 'Lys-119', rendering chromatin heritably changed in its expressibility (PubMed:25519132). The PRC1-like

	complex that contains PCGF5, RNF2, CSNK2B, RYBP and AUTS2 has decreased histone H2A ubiquitination activity, due to the phosphorylation of RNF2 by CSNK2B (PubMed:25519132). Consequently, the complex mediates transcriptional activation (PubMed:25519132). In the cytoplasm, plays a role in axon and dendrite elongation and in neuronal migration during embryonic brain development. Promotes reorganization of the actin cytoskeleton, lamellipodia formation and neurite elongation via its interaction with RAC guanine nucleotide exchange factors, which then leads to the activation of RAC1 (By similarity).
**Location:**<br>71,132,144-<br>71,713,599<br>forward strand	GALNT17, polypeptide N-acetylgalactosaminyltransferase 17: **(581,456 nt)**<br><br>**Biased expression in brain (RPKM 15.1 ±7,12),** ovary (RPKM 6.4) and 12 other tissues.<br>This gene encodes an N-acetylgalactosaminyltransferase. This gene is located centromeric to the common deleted region in Williams-Beuren syndrome (WBS), a multisystem developmental disorder caused by the deletion of contiguous genes at 7q11.23. This protein may play a role in membrane trafficking.<br>**GeneCards:** Diseases associated with GALNT17 include Williams-Beuren Syndrome and Autism Spectrum Disorder. Among its related pathways are Metabolism and Metabolism of proteins. An important paralog of this gene is GALNT9 (chr:12q24.33).<br>**Prot-Summary:** May catalyze the initial reaction in O-linked oligosaccharide biosynthesis, the transfer of an N-acetyl-D-galactosamine residue to a serine or threonine residue on the protein receptor.
